# Abemaciclib induces G1 arrest and lysosomal dysfunction in canine melanoma cells: synergistic effects with fenbendazole

**DOI:** 10.3389/fvets.2025.1603686

**Published:** 2025-06-26

**Authors:** Se-Hoon Kim, Jun-Yeol Choi, Yoon-Ho Suh, Ki-Hoon Song, Min-Ok Ryu, Kyoung-Won Seo

**Affiliations:** ^1^Laboratory of Veterinary Internal Medicine, Department of Veterinary Clinical Sciences, College of Veterinary Medicine, Seoul National University, Seoul, Republic of Korea; ^2^Research Institute, ViroCure Inc., Seoul, Republic of Korea

**Keywords:** autophagy, CDK4/6 inhibitor, combination therapy, G1 arrest, lysosomal dysfunction, senescence, vacuolization

## Abstract

**Introduction:**

Abemaciclib, a CDK4/6 inhibitor, is well-established for treating hormone receptor-positive and HER2-negative (HR^+^/HER2^−^) human breast cancer by inducing G1 cell cycle arrest. However, its antitumor effects in other cancers, including canine melanoma, remain largely unexplored. Canine melanoma often harbors CDK4/6 copy number gains and cell cycle dysregulation, suggesting it may be a suitable target for abemaciclib.

**Methods:**

Five canine melanoma cell lines (CMeC1, KMeC, LMeC, UCDK9M4, and UCDK9M5) were used to evaluate the antitumor effects of abemaciclib. Cell viability and migration were assessed using Cell Counting Kit-8 and wound healing assays, respectively. Cell cycle distribution was analyzed by flow cytometry, and the expression of cell cycle-related genes and proteins was examined using RT-PCR and western blotting. The origin of cytoplasmic vacuoles was investigated using FITC-dextran uptake and V-ATPase inhibitor assays. Impaired autophagic flux was assessed by immunofluorescence detection of p62 accumulation. Synergistic effects with fenbendazole were evaluated using the Highest Single Agent (HSA) synergy scoring method, and *in vivo* efficacy was assessed in a xenograft model.

**Results:**

Abemaciclib induced G1 cell cycle arrest and altered the expression of cell cycle-related genes and proteins. Autophagy, but not apoptosis, was activated. Vacuolization was observed and suggested to originate from lysosomes, as evidenced by FITC-dextran uptake and V-ATPase inhibitor co-treatment. p62 accumulation indicated impaired autophagy flux. Co-treatment with fenbendazole enhanced cytotoxicity and showed synergistic effects within specific dose ranges. *In vivo*, abemaciclib alone or in combination with fenbendazole significantly suppressed tumor growth.

**Discussion:**

These findings demonstrate that abemaciclib exerts potent antitumor effects in canine melanoma by inducing cell cycle arrest and disrupting lysosomal function. Its synergistic interaction with fenbendazole suggests a potential combinatorial therapeutic approach for canine melanoma.

## 1 Introduction

Canine malignant melanoma is a relatively common neoplasm in dogs and originates primarily in the oral cavity, eyes, digits, and skin (1–3). Multimodal therapies, including surgery, chemotherapy, immunotherapy, electrochemotherapy, and radiotherapy are used to treat canine malignant melanoma (4). Loco-regional control of canine malignant melanoma using surgery or radiotherapy can also be effective (5). However, treating disseminated disease with cytotoxic drugs has shown limited success, with minimal improvement in survival time (5). Canine malignant melanoma is characterized by immunogenicity and chromosomal instability, often associated with abnormalities in the spindle assembly checkpoint (1, 6, 7). A recent genetic study categorized canine malignant melanomas into low (hot immune) and high (cold immune) structural variant groups. For tumors in the high structural variant group, targeting amplified oncogenes using CDK4/6 inhibitors may provide effective treatment options (8). The cyclin-dependent kinase (CDK)-RB1-E2F axis plays a crucial role in cell division and cycle regulation (9, 10). The chromatin-associated RB protein is a key cell cycle controller (11). When RB binds to E2F, transcription is suppressed. However, RB phosphorylation by CDK-cyclin complexes causes it to detach from E2F proteins, allowing E2F target gene transcription. Dysregulation of this axis can result in uncontrolled cell proliferation (9, 12).

CDK4/6 inhibitors such as palbociclib, ribociclib, and abemaciclib are approved by the Food and Drug Administration and European Medicines Agency for use in breast cancer (HR^+^/HER2^−^) when combined with hormonal therapeutics (13). Since CDK4-RB1-E2F pathway dysregulation is frequently observed in multiple human cancer types, these inhibitors likely have applications beyond breast cancer (14, 15). Notably, CDK4/6 pathway alterations are found in up to 90% of human melanomas (16), and CDK4 copy number changes have been reported in canine melanoma (8, 17). Although not approved for other cancers, studies are underway to explore their efficacy in melanoma, both as standalone treatments and in combination with other therapies (16, 18).

Lysosomes are membrane-enclosed organelles containing acidic hydrolase enzymes (19) and serve as the primary degradative compartments of the cell. They are essential for various physiological processes, including autophagy, phagocytosis, exosome release, antigen processing, lysosomal cell death induction, and cellular signaling regulation (20). Lysosomal acidity (pH 4.5–5.0) is maintained by vacuolar-type ATPases (V-ATPases), which actively pump protons into the lumen (21). Impaired lysosomal function due to defects in enzymes or V-ATPase subunits, is associated with lysosomal storage diseases (22) and neurodegenerative disorders such as Parkinson’s disease (23) and Alzheimer’s disease (24).

Autophagy is a highly conserved cellular degradation and recycling process across all eukaryotic organisms (25, 26), and was first identified in the 1960s in mammalian cells (27). It is classified into microautophagy, macroautophagy, and chaperone-mediated autophagy (25), with macroautophagy being the most widespread. Macroautophagy comprises induction, elongation, maturation, fusion, and degradation (25). Following induction, a phagophore is formed and elongates to become autophagosome. Autophagosome maturation includes several fusion events with early and late endosomes, as well as lysosomes, and it allows convergence of the endocytic and autophagic pathways (28). Subsequently, autophagosomes fuse with lysosomes to generate autolysosomes (28). Following fusion, the autophagic body is likely degraded by lytic enzymes present within the lysosomal lumen (27).

Abemaciclib has distinct features when compared with other CDK4/6 inhibitors. Abemaciclib readily inactivates the CDK4/6 ATP-binding pocket due to its small substituent (10, 29). Among CDK4/6 inhibitors, abemaciclib is the most potent, demonstrating five times greater potency for CDK4 than CDK6 (10). Additionally, it has fewer hematologic side effects than palbociclib and has higher gastrointestinal effects (30). Abemaciclib induces an unusual form of cell death, characterized by cytoplasmic vacuolization (31). In veterinary medicine, studies on palbociclib have demonstrated its chemotherapeutic effects in canine mammary tumors and melanoma. However, other CDK4/6 inhibitors, including abemaciclib, remain largely unexplored (17, 32). Given the success of CDK4/6 inhibitors in human medicine, their application in veterinary medicine, particularly that of abemaciclib, remains limited. This study aimed to evaluate the chemotherapeutic and cell cycle arrest effects of abemaciclib in canine malignant melanoma, as well as its potential synergy with fenbendazole.

## 2 Methods

### 2.1 Cell culture and cell line authentication statement

Five canine malignant melanoma cell lines were selected for this study: four from the oral cavity (UCDK9M4, UCDK9M5, KMeC, and LMeC) and one from the skin (CMeC1). UCDK9M4 and KMeC were derived from primary oral tumors, and UCDK9M5 originated from lymph node metastasis from primary oral tumors, whereas LMeC originated from a lymph node metastasis (33, 34). KMeC, LMeC, and CMeC1 were kindly provided by Dr. Takayuki Nakagawa, whereas UCDK9M4 and UCDK9M5 were kindly provided by Dr. Michael Kent. Canine skin fibroblasts were generously provided by Dr. Minkyu Kim.

UCDK9M4, UCDK9M5, CMeC1, and KMeC were cultured in Dulbecco’s Modified Eagle Medium (DMEM; SolBio, Suwan, Korea; Cat. #DME-001) with 10% fetal bovine serum (FBS; Gibco, Thermo Fisher, Waltham, MA, United States; Cat. #16000-044), whereas LMeC cells were cultured Roswell Park Memorial Institute (RPMI)-1640 medium (SolBio, Suwan, Korea; Cat. #RPM-001) supplemented with 10% FBS. All cells were incubated at 37°C in a humidified atmosphere with 5% CO_2_. Abemaciclib was purchased from Selleck Chemicals (Houston, TX, United States; Cat. #S5716) and dissolved in dimethyl sulfoxide (DMSO; Sigma-Aldrich; Cat. #D2650). The highest DMSO concentrations were 0.25% for cell viability assays, 0.056% for western blot, and 0.05% for cell cycle analysis.

### 2.2 Cell viability assay and combination studies

Cell viability was evaluated using the Cell Counting Kit-8 (CCK-8) assay (DOJINDO Laboratories, Kumamoto, Japan, #CK04). Cells were seeded at a density of 2,000 cells/well in 96-well plates and incubated overnight. The medium was replaced with serum-free medium for 12 h to synchronize the cells, followed by treatment with abemaciclib at varying concentrations for 72 h. Afterward, the medium was replaced with fresh medium and 10 μL of CCK-8 reagent was added and incubated at 37°C for 3 h. Absorbance was measured at 450 nm using a microplate reader (ELx800, Biotek Instruments, Winooski, VT, United States). Cell viability was calculated as a percentage of control values using the following formula: Cell viability (% of control cells) = [(Optical density (OD) of treated sample − OD of blanks)]/[(OD of control sample − OD of blanks)] × 100%. Experiments were conducted in duplicate and repeated thrice. Half-maximal inhibitory concentration (IC_50_) values were calculated using GraphPad Prism 10.1.2 (GraphPad Software, San Diego, CA, United States). For combination studies of abemaciclib and fenbendazole (Sigma-Aldrich, Burlington, MA, United States; Cat. #F5396), cell viability data were analyzed using SynergyFinder Plus software with the HSA model (35).

### 2.3 Colony formation assay

A colony formation assay was performed by seeding 500 cells per well in 6-well plate and incubating for 8 h prior to treatment with various abemaciclib concentrations. Treatment medium was replaced every 3 days and incubated for 8 days. Colonies were stained with 0.5% crystal violet solution in methanol.

### 2.4 Cell staining and morphology

Cells were seeded on coverslips in 35-mm dishes and allowed to adhere for 12 h before treatment. Following 24 h treatment with various concentrations of abemaciclib, cells were fixed and stained with Diff-Quik (Sysmex Asia Pacific, Woodlands Spectrum, Singapore; Cat. #38721). Images were captured at a magnification of 1,000× using a digital microscope (Eclipse E200, Nikon, Tokyo, Japan).

### 2.5 Wound healing assay

Cells were seeded at 1 × 10^6^ cells per well in 6-well plates and grown to 100% confluence. A scratch was created on the monolayer using a sterilized 200 μL micropipette tip. Wells were washed with medium to remove debris and non-adherent cells. Cells were then treated with various concentrations of abemaciclib. Images of wound closure were captured at 0, 6, 12, and 24 h (CMeC1, KMeC, LMeC, and UCDK9M4) and additional time points of 30 and 36 h (UCDK9M5). Wound areas were analyzed using Fiji ImageJ software and Wound Healing Size Tool (36, 37). Wound closure was calculated as: Wound area (%) = (Wound area at time zero − Wound area at time *A*)/(Wound area at time zero) × 100, where *A* represents the time after scratch induction.

### 2.6 Cell cycle analysis

Cells (3 × 10^5^) were seeded in 60-mm dishes with complete medium and incubated overnight. Media was replaced with FBS-free media and incubated for 24 h for serum starvation and cell cycle synchronization. The melanoma cells were treated with DMSO, 0.5, 1, 2, or 4 μM of abemaciclib for 24 h. Attached cells were harvested with trypsin and fixed with 70% cold ethanol, then incubated overnight at −20°C. Fixed cells were washed with phosphate buffered saline (PBS), and 1 × 10^5^ cells were collected and stained with 0.5 mL of propidium iodide/RNase staining buffer (BD Transduction Laboratories, San Diego, CA, United States, #550825). Flow cytometry was performed using MACSQuant Analyzer 10 (Miltenyi Biotec, Bergisch Gladbach, Germany) with FlowJo software (BD Biosciences, Ashland, OR, United States). At least 10,000 events were collected for each sample, and data were analyzed as percentages of G1, S, and G2/M phases.

### 2.7 Protein extraction and western blotting

Proteins were extracted using radioimmunoprecipitation assay (RIPA) buffer supplemented with protease and phosphatase inhibitors (SmartGene, Daejeon, Korea; Cat. #SG-PR-CELI). After incubation on ice, cell lysates were centrifuged at 10,000 × *g* at 4°C for 20 min, and supernatants were collected. Standard curve obtained from bicinchoninic acid assay (Thermo Fisher, Waltham, MA, United States; Cat. #23227) was utilized to calculate protein concentration. Diluted samples with sample buffers (SmartGene 5X Sample buffer, SamJung Bioscience, Daejeon, Korea; Cat. #SG-PR-SB) were heated for 5 min at 100°C. Samples containing 10 μg protein were separated via sodium dodecyl sulfate-polyacrylamide gel electrophoresis (SDS-PAGE) and transferred to nitrocellulose membranes (ATTO, Tokyo, Japan; Cat. #AE-6667-N). After blocking with 5% bovine serum albumin in TBST, membranes were probed overnight at 4°C with the primary antibodies listed in Supplementary Table 1, followed by incubation with HRP-conjugated secondary antibodies for 1 h at 21°C. Chemiluminescent signals were visualized using the SmartGene ECL High Femto Solution (SmartGene, Daejeon, Korea; Cat. #SG-PR-HECL) and a Las 4,000 imager (GE Healthcare Biosciences, Uppsala, Sweden).

### 2.8 Dextran uptake assay

Melanoma cells were seeded in 35-mm confocal dishes and incubated for 12 h. The medium was replaced with serum-free medium and incubated for another 12 h. Subsequently, the cells were stained with 1 μg/mL Hoechst 33342 (BDL Pharmatech, Shanghai, China; Cat. #BD133718) for 15 min, followed by three washes with medium. Cells were then treated with 2.5 μM abemaciclib or control media containing 0.2 mg/mL FITC-dextran (Sigma-Aldrich, Burlington, MA, United States; Cat. #FD70S) under serum-free conditions. After 12 h, cells were fixed with 4% paraformaldehyde, and fluorescent images were captured using an EVOS 2FL imaging system (Thermo Fisher, Waltham, MA, United States).

### 2.9 Vacuolization inhibition

To investigate the origin of vacuoles, bafilomycin A1 (MedChemExpress, Monmouth Junction, NJ, United States; Cat. #HY-100558), concanamycin A (MedChemExpress; Cat. #HY-N1724), or cycloheximide (Tocris Bioscience, Bristol, United Kingdom; Cat. #0970) was added 2 h prior to abemaciclib treatment and co-incubated with 2.5 μM abemaciclib for 12 h. Images were captured using a phase-contrast microscope.

### 2.10 Quantitative real-time RT-PCR

Quantitative real-time polymerase chain reaction (RT-PCR) was performed to analyze the mRNA expression levels of genes involved in various stages of proliferation. Each cell line was treated for 12 h, and the mRNA expression levels of CCNA2, CCNB1, CCNE1, CCNE2, CCND1, CDK4 and CDK6 were analyzed. Primers were designed using OligoPerfect Designer (Thermo Fisher Scientific). Each primer was validated by generating a standard curve to calculate amplification efficiency, and its specificity was confirmed using NCBI blast. Primer sequences used for amplification are listed in Supplementary Table 2.

Total RNA was extracted using Easyblue reagent (iNtRON Biotechnology, Yongin, Korea; Cat. #17061) and reverse-transcribed into cDNA using the Compact cDNA Synthesis Kit (Smartgene, Daejeon, Korea; Cat. #SG-cDNAC100). RT-PCR was performed with SmartGene SYBER Green Q-PCR Master Mix (Smartgene; Daejeon, Korea; Cat. #SG-SYBR-ROXL02), with GAPDH as internal control. Relative mRNA expression was calculated using the 2^−∆∆Cq^ method. Each reaction was duplicated, and the experiment was independently repeated thrice.

### 2.11 Immunofluorescence analysis

To validate the antibody specificity and exclude non-specific binding or autofluorescence, cells were incubated with secondary antibodies alone, without primary antibodies.

Cells (2.4 × 10^4^) were seeded in 24-well plates on Ø 12-mmcoverslips (Marienfeld Superior, Lauda-Königshofen, Germany; Cat. #HSU-011520) and incubated for 12 h for cell attachment. Each group was treated for 24 h as follows: for the negative control, serum starvation was applied; the treatment group received abemaciclib (0.5, 2.5, and 5 μM); and bafilomycin A1 (100 nM) was used as positive control.

After treatment, cells were washed thrice with cold PBS and fixed with 4% paraformaldehyde (T&I Biotechnology Co., Ltd., Hsinchu, Taiwan; Cat. #BPP-9004) for 5 min at 21°C. Subsequently, cells were washed with PBS for 5 min, thrice with gentle agitation. For permeabilization, cells were treated with 0.3% Triton X-100 in PBST for 10 min at 21°C, followed by washing with PBS. Blocking was performed using 3% BSA in PBST containing 0.3 M glycine for 30 min at 21°C. After washing with PBS, cells were incubated overnight at 4°C with primary antibodies against p62 (Abclonal, Cat. #A19700; 1:200) and β-tubulin (Abclonal, Cat. #AC012; 1:200), diluted in PBST containing 3% BSA. The following day, cells were washed with PBS and incubated with the secondary antibodies: FITC-conjugated anti-rabbit IgG (Abclonal, Cat. #AS011; 1:200) and Tetramethylrhodamine (TRITC)-conjugated anti-mouse IgG (Abclonal, Cat. #AS026; 1:200) for 1 h in the dark. Following staining, the coverslips were mounted onto glass slides using Fluoromount-G mounting medium with DAPI (Invitrogen, Carlsbad, CA, United States; Cat, #00-4958-02), and fluorescence images were acquired using a confocal microscope (LSM 800, Carl Zeiss, Oberkochen, Germany).

### 2.12 *In vivo* study

The UCDK9M5 cell line was selected based on *in vitro* results demonstrating the highest sensitivity and synergistic response to abemaciclib and fenbendazole treatment. A preliminary pilot study was conducted using three mice to optimize tumor establishment, determine appropriate number of cells for injection, and estimate the tumor growth period. Based on previous *in vivo* studies using similar xenograft models, the number of mice per group was set to the minimum required to obtain statistically meaningful results while minimizing animal use (38).

Forty female BALB/_C_ nude mice (6 weeks old) were subcutaneously inoculated with 5 × 10^6^ UCDK9M5 cells into the right rear flank. When the mean tumor volume reached 100–150 mm^3^, mice exhibiting extreme tumor volume variations (either excessively large or small) were euthanized. The remaining mice were then randomized into control and treatment groups, with seven mice assigned to each group. As reported in previous studies (38), the drugs were prepared in 1% hydroxyethyl cellulose dissolved in 25 mmol/L phosphate buffer (pH 2) and administered orally by gavage once daily (final volume: 0.2 mL). Fenbendazole, which could not be fully dissolved in the vehicle, was administered as a suspension. Although it remained insoluble, the suspension was thoroughly mixed prior to each administration to ensure consistent dosing.

The dose of abemaciclib and fenbendazole was both 50 mg/kg. Body weight and tumor volumes (length × width^2^ × 0.5) were measured every 2 days. Mice were euthanized when tumor volumes reached 2,000 mm^3^. All animal experiments were performed in accordance with the guidelines of Institutional Animal Care and Use Committee (IACUC) and approved under protocol number SNU-240614-1.

### 2.13 Statistical analysis

GraphPad Prism 10.1.2 was used for all analyses. The Kruskal–Wallis test with Dunn’s *post hoc* test was applied for comparisons. Data are expressed as mean ± standard deviation, with *p* value < 0.05 was considered significant.

## 3 Results

### 3.1 Abemaciclib reduces canine melanoma cell viability and alters morphology

**Figure 1:**
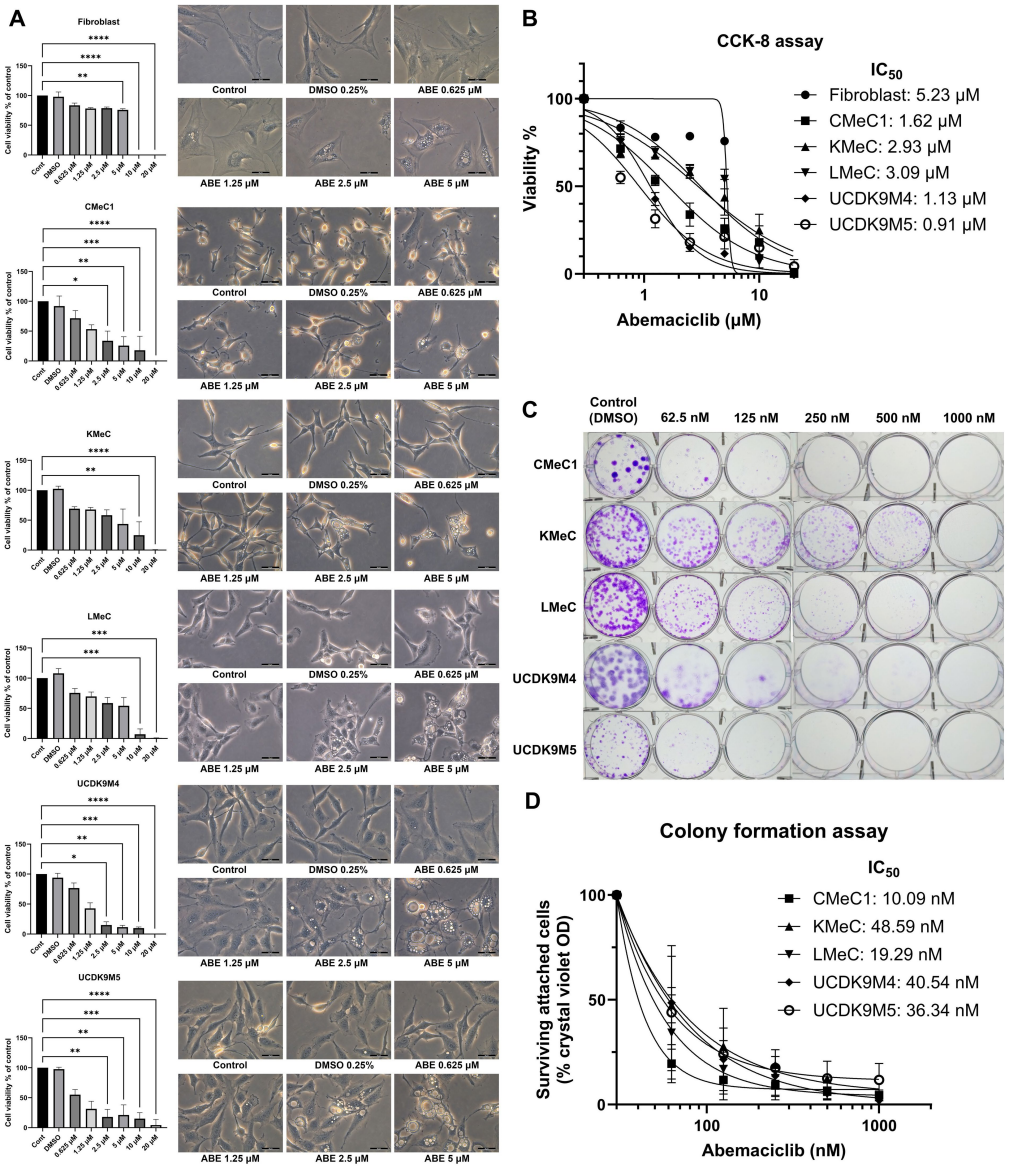
Effects of abemaciclib on cell viability, proliferation, and morphological changes in canine melanoma cell lines. **(A)** Cell viability and morphological changes following abemaciclib treatment. Canine melanoma cell lines (CMeC1, KMeC, LMeC, UCDK9M4, and UCDK9M5) and fibroblasts were treated with increasing concentrations of abemaciclib (0.625, 1.25, 2.5, 5, 10, and 20 μM) for 72 h. Cell viability was analyzed using the Cell Counting Kit-8 assay, and morphological changes were imaged after 24 h of treatment. Bar graphs represent cell viability relative to the control (mean ± SD; ^*^p < 0.05, ^**^p < 0.01, ^***^p < 0.001, and ^****^p < 0.0001). Representative phase-contrast images illustrate dose-dependent morphological changes, including cell flattening and vacuolization following abemaciclib treatment. Scale bar = 50 μm. **(B)** Dose-response curves and IC_50_ values of abemaciclib. Cell viability was measured across different concentrations of abemaciclib, and IC_50_ values were calculated for each cell line and fibroblasts. Melanoma cell lines exhibited lower IC_50_ values than fibroblasts, indicating higher sensitivity to abemaciclib. **(C)** Colony formation assay. Canine melanoma cell lines (CMeC1, KMeC, LMeC, UCDK9M4, and UCDK9M5) were treated with increasing concentrations of abemaciclib (62.5, 125, 250, 500, and 1,000 nM) for 8 days. Representative images of colonies stained with crystal violet demonstrate dose-dependent inhibition of colony formation. **(D)** Quantification of colony formation using crystal violet absorbance. Colonies stained with crystal violet in the colony formation assay were solubilized in methanol, and absorbance was measured to determine relative cell survival (% of control).

The cell viability assay revealed a dose-dependent reduction in viability with abemaciclib treatment (Figure 1A). To evaluate toxicity in normal mesenchymal cells, canine skin fibroblasts were used as a reference. Fibroblast viability significantly decreased at 5 μM and showed a marked reduction at 10 μM. In contrast, significant reductions in cell viability were observed in canine melanoma cell lines. CMeC1, UCDK9M4, and UCDK9M5 exhibited significant decrease starting at 2.5 μM, while LMeC and KMeC showed significant reductions beginning at 10 μM. The calculated IC_50_ values for abemaciclib were as follows: fibroblasts, 5.23 μM; CMeC1, 1.62 μM; KMeC, 2.93 μM; LMeC, 3.09 μM; UCDK9M4, 1.13 μM; and UCDK9M5, 0.91 μM (Figure 1B). Fibroblasts exhibited the highest IC_50_ value, indicating lower sensitivity compared to all melanoma cell lines, while UCDK9M5 and UCDK9M4 were the most sensitive, with IC_50_ values of 0.91 μM and 1.13 μM, respectively.

A colony formation assay was performed to evaluate the long-term effects of abemaciclib (Figures 1C, D). No colony formation was observed in any of the canine melanoma cell lines at 1,000 nM. Although KMeC and LMeC showed reduced inhibition of colony formation, notable inhibition was still observed, even at a concentration as low as 62.5 nM. Morphological changes, such as cell flattening, enlargement, and vacuolization, were identified at 2.5 μM and became prominent at 5 μM (Figure 1A; Supplementary Figure 1).

### 3.2 Abemaciclib suppresses cell migration

**Figure 2:**
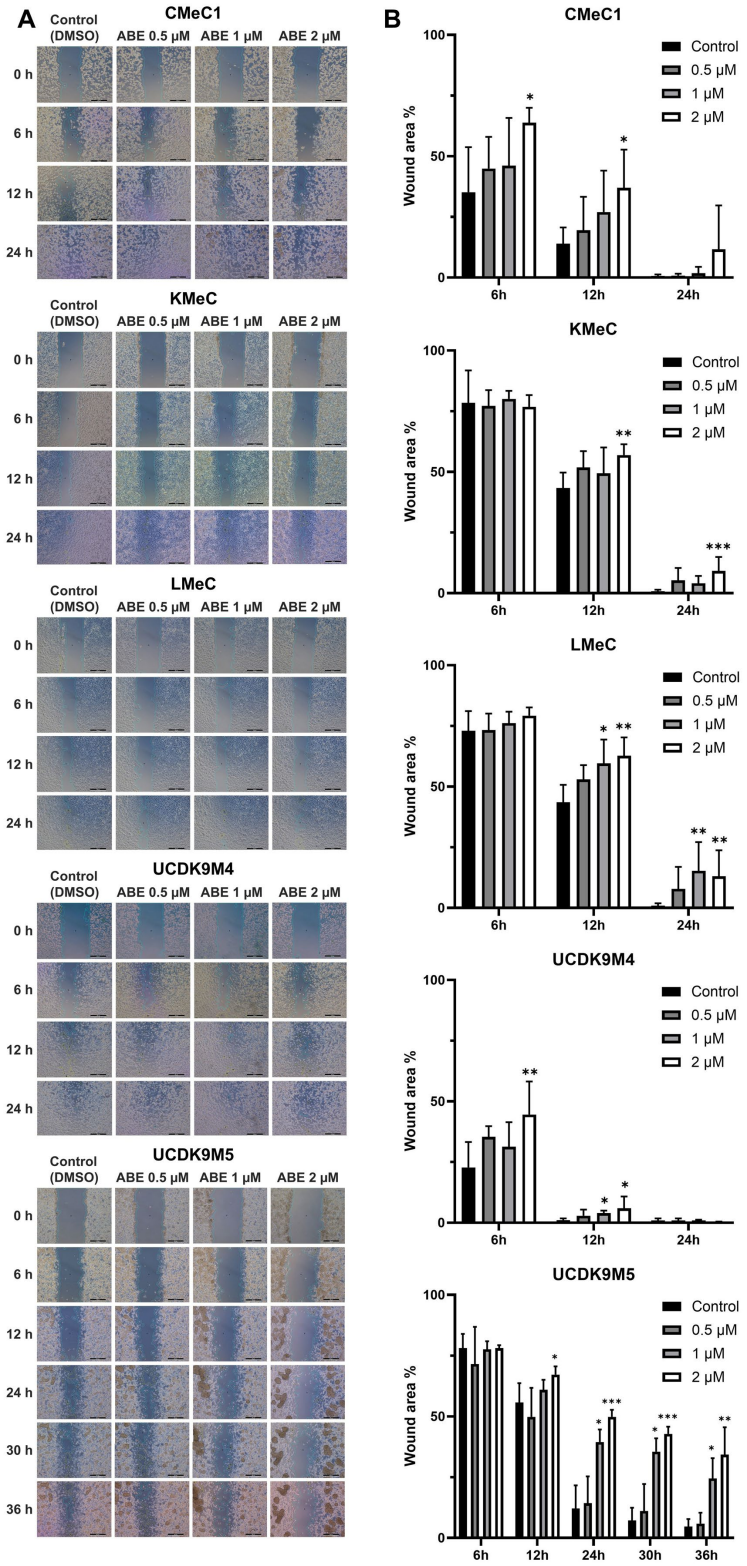
Abemaciclib inhibits wound healing in canine melanoma cell lines. **(A)** Representative images of wound healing assays. Canine melanoma cell lines (CMeC1, KMeC, LMeC, UCDK9M4, and UCDK9M5) were treated with abemaciclib at 0.5, 1, or 2 μM. Wound closure was monitored at 0, 6, 12, and 24 h for CMeC1, KMeC, LMeC, and UCDK9M4, and up to 36 h for UCDK9M5. Control cells were treated with DMSO (vehicle). The green line indicates the measured wound area. The images demonstrate a dose-dependent inhibition of wound closure with abemaciclib treatment. Scale bar = 500 μm. **(B)** Quantitative analysis of the wound area. The wound area percentage was measured at the indicated time points (6, 12, 24, 30, and 36 h) relative to the initial wound size. Bar graphs represent the mean ± SD (*n* = 6), obtained from two replicates across three independent experiments. (^*^p < 0.05, ^**^p < 0.01, and ^***^p < 0.001).

The migration ability of canine melanoma cells was assessed following treatment with abemaciclib at concentrations at 0.5, 1, and 2 μM (Figure 2A). CMeC1, KMeC, LMeC, and UCDK9M4 cells achieved nearly complete wound closure within 24 h, whereas UCDK9M5 cells required more than 36 h. In CMeC1 cells, migration was effectively inhibited by abemaciclib at 2 μM after 6 and 12 h (Figure 2B). In KMeC cells, significant inhibition was observed at 12 and 24 h with 2 μM. In LMeC cells, migration was suppressed at 12 and 24 h at 1 μM and 2 μM. Similarly, in UCDK9M4 cells, abemaciclib demonstrated inhibitory effects at 6 h at 2 μM, and at 12 h at 1 and 2 μM. Finally, in UCDK9M5 cells, abemaciclib significantly inhibited migration at 12 h at 2 μM, and at 24, 30, and 36 h at 1 and 2 μM.

### 3.3 Abemaciclib induces G1 arrest

**Figure 3:**
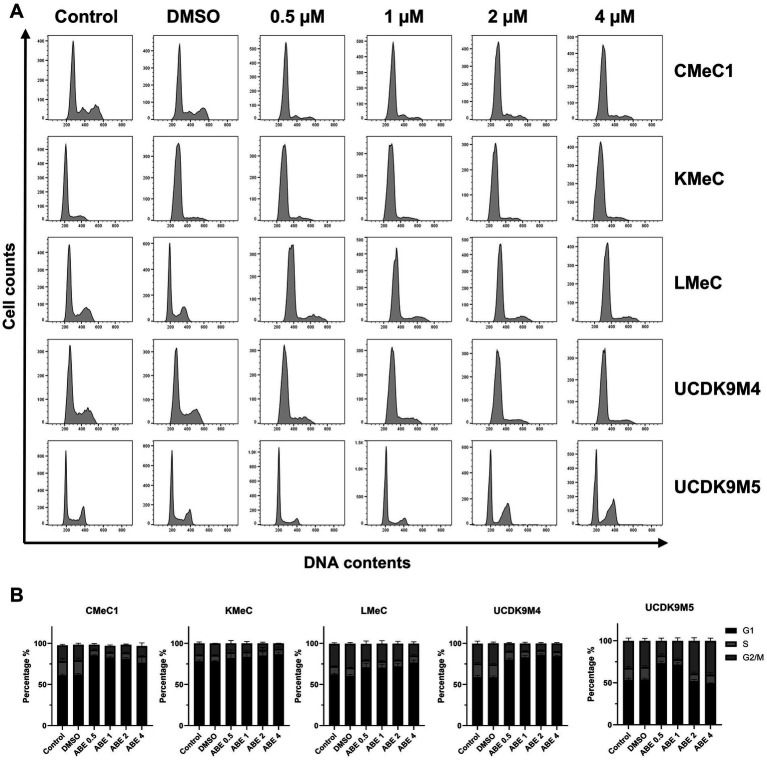
Effects of abemaciclib on the cell cycle distribution. **(A)** Flow cytometry analysis of cell cycle progression was performed in five different cell lines (CMeC1, KMeC, LMeC, UCDK9M4, and UCDK9M5) after treatment with increasing concentrations of abemaciclib (0.5, 1, 2, and 4 μM) for 24 h. Control and DMSO-treated groups were included as baselines. The histograms show DNA contents (x-axis) versus cell count (y-axis), indicating the proportion of cells in different phases of the cell cycle (G1, S, and G2/M). **(B)** Quantification of cell cycle phases (G1, S, and G2/M) was performed using flow cytometry data. The percentage of cells in each phase is presented for all five cell lines (CMeC1, KMeC, LMeC, UCDK9M4, and UCDK9M5) treated with abemaciclib. Abemaciclib treatment induces a concentration-dependent G1 phase arrest across most cell lines, except for UCDK9M5 2 and 4 μM treatment, where this effect is reduced.

Since abemaciclib inhibits CDK4/6, it induces G1 cell cycle arrest. FACS was used to evaluate this effect, and the results confirmed that abemaciclib induced G1 cell cycle arrest, which became prominent at concentrations of 1 μM or higher (Figures 3A,B). In UCDK9M5, G1 arrest was initiated at 0.5 μM, while a notable increase in the G2/M population was observed at 2 μM and higher concentrations.

### 3.4 Abemaciclib regulates cell cycle and induces autophagy in canine melanoma cells

**Figure 4:**
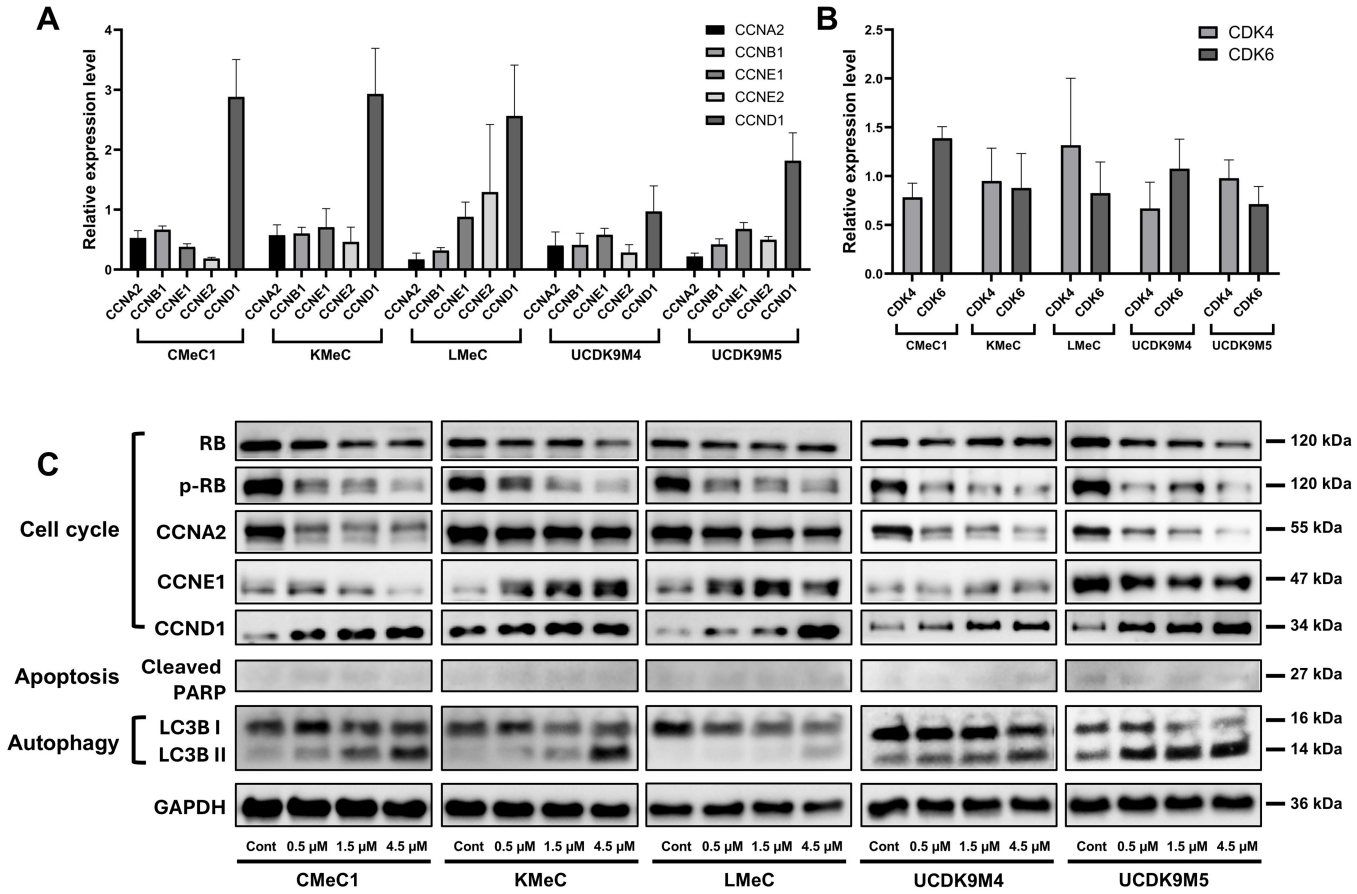
Effects of abemaciclib treatment on cell cycle regulation, apoptosis, and autophagy-regulated protein expression. **(A)** Relative mRNA expression levels of cell cycle-related genes (CCNA2, CCNB1, CCNE1, CCNE2, and CCND1) were measured following treatment with abemaciclib at 1 μM for 12 h. Data are presented as mean ± SD (*n* = 6), obtained from two replicates across three independent experiments. **(B)** Relative mRNA expression levels of CDK4 and CDK6 under the same treatment conditions. **(C)** Western blot analysis showing protein expression levels of key regulators of the cell cycle (RB, p-RB, CCNA2, CCNE1, and CCND1), apoptosis (cleaved PARP), and autophagy (LC3B I/II). Cells were treated with increasing concentrations of abemaciclib for 24 h, and GAPDH was used as a loading control.

To elucidate the abemaciclib mechanism of action, we analyzed the relative RNA levels of cyclins and CDKs, as well as protein levels of cell cycle regulators, apoptosis-related markers, and autophagy-related markers (Figures 4A–C). According to the RT-PCR results, CCNA2 and CCNB1 expression levels decreased across all canine melanoma cell lines, whereas CCND1 expression was notably increased. CCNE1, CCNE2 levels were relatively low in CMeC1, KMeC, UCDK9M4, and UCDK9M5, but CCNE2 was markedly elevated in LMeC. CDK4 and CDK6 were expressed in all cell lines, but the levels differed between cell lines without showing a consistent trend.

In the western blot analysis, abemaciclib downregulated cell cycle regulatory proteins, including phosphorylated RB (p-RB) and Cyclin A2, but increased the levels of Cyclin D1 (Figure 4C). Regarding RB, most cell lines showed decreased expression following treatment, whereas UCDK9M4 cells exhibited a slight increase. Cyclin E1 expression had varying trends: it increased in KMeC and LMeC cells but decreased in CMeC1, UCDK9M4, and UCDK9M5 cells. The apoptosis-related marker, cleaved PARP, exhibited weak signal intensity across all cell lines, suggesting that abemaciclib treatment did not strongly induce apoptosis under these conditions. In contrast, autophagy markers demonstrated a clear trend, with LC3B-I levels decreasing and LC3B-II levels increasing in all cell lines, indicating autophagy induction.

### 3.5 Abemaciclib-induced vacuolization and its inhibition by V-ATPase inhibitors

**Figure 5:**
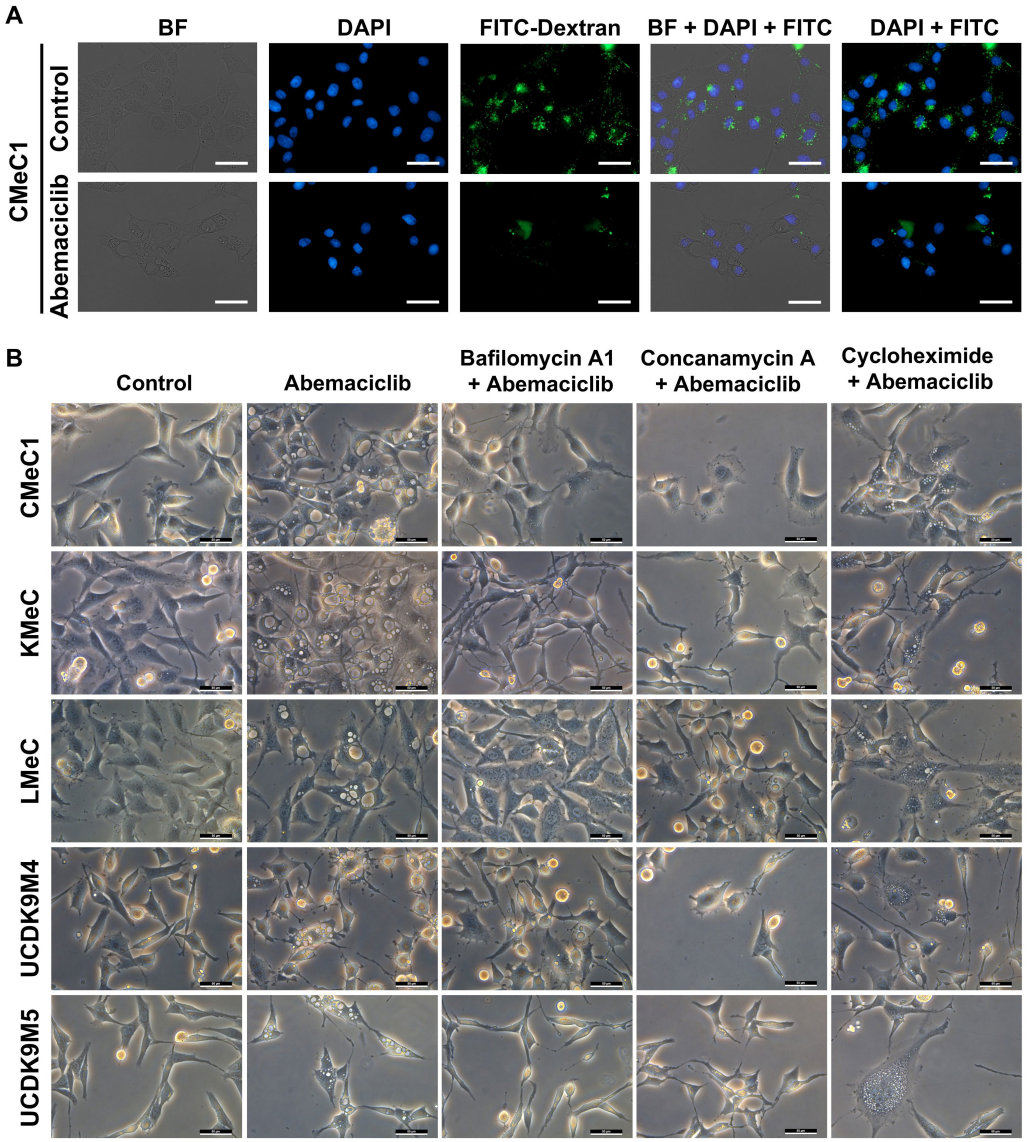
Dextran uptake assay and vacuolization inhibition test with V-ATPase inhibitor and protein synthesis inhibitor. **(A)** Dextran uptake assay. Under serum starvation conditions, CMeC1 control cells exhibited uptake of FITC-Dextran (green) through pinocytosis. However, treatment with 2.5 μM abemaciclib led to vacuole formation in the cytoplasm, but these vacuoles did not contain FITC-Dextran, suggesting that the vacuoles originated from intracellular components rather than extracellular uptake. Nuclei were stained with DAPI (blue). Images include bright-field (BF), DAPI, FITC-Dextran, DAPI merged with FITC, and BF merged with DAPI and FITC. Scale bar = 50 μm. **(B)** Vacuolization inhibition test with V-ATPase inhibitors (bafilomycin A1 and concanamycin A) and a protein synthesis inhibitor (cycloheximide). Cells were pre-treated with inhibitors (bafilomycin A1 20 nM, concanamycin A 10 nM, or cycloheximide 2 μM) for 2 h prior to abemaciclib treatment and observed after 12 h. Bafilomycin A1 and concanamycin A effectively suppressed vacuolization at 12 h. Cycloheximide partially suppressed vacuole formation at 12 h. Scale bar = 50 μm. BF, bright field.

To determine whether vacuoles originated from extracellular components, a FITC-dextran assay under serum starvation conditions was conducted to promote pinocytosis. Control cells exhibited pinocytosis, as indicated by FITC fluorescence in the cytoplasm (Figure 5A). However, treatment with 2.5 μM abemaciclib inhibited pinocytosis and induced vacuole formation. Notably, FITC-dextran was not detected within the vacuoles, suggesting that the vacuoles did not arise from extracellular uptake. To further explore the origin of vacuoles, we tested V-ATPase and protein synthesis inhibitors (Figure 5B). Treatment with bafilomycin A1 (20 nM) or concanamycin A (10 nM) for 12 h effectively suppressed vacuolization. Cycloheximide (2 μM), a protein synthesis inhibitor that suppresses paraptosis, reduced vacuole formation but did not completely inhibit it. These findings suggest that vacuoles may arise from an intracellular process regulated by V-ATPase activity.

### 3.6 Autophagy completion inhibition by abemaciclib

**Figure 6:**
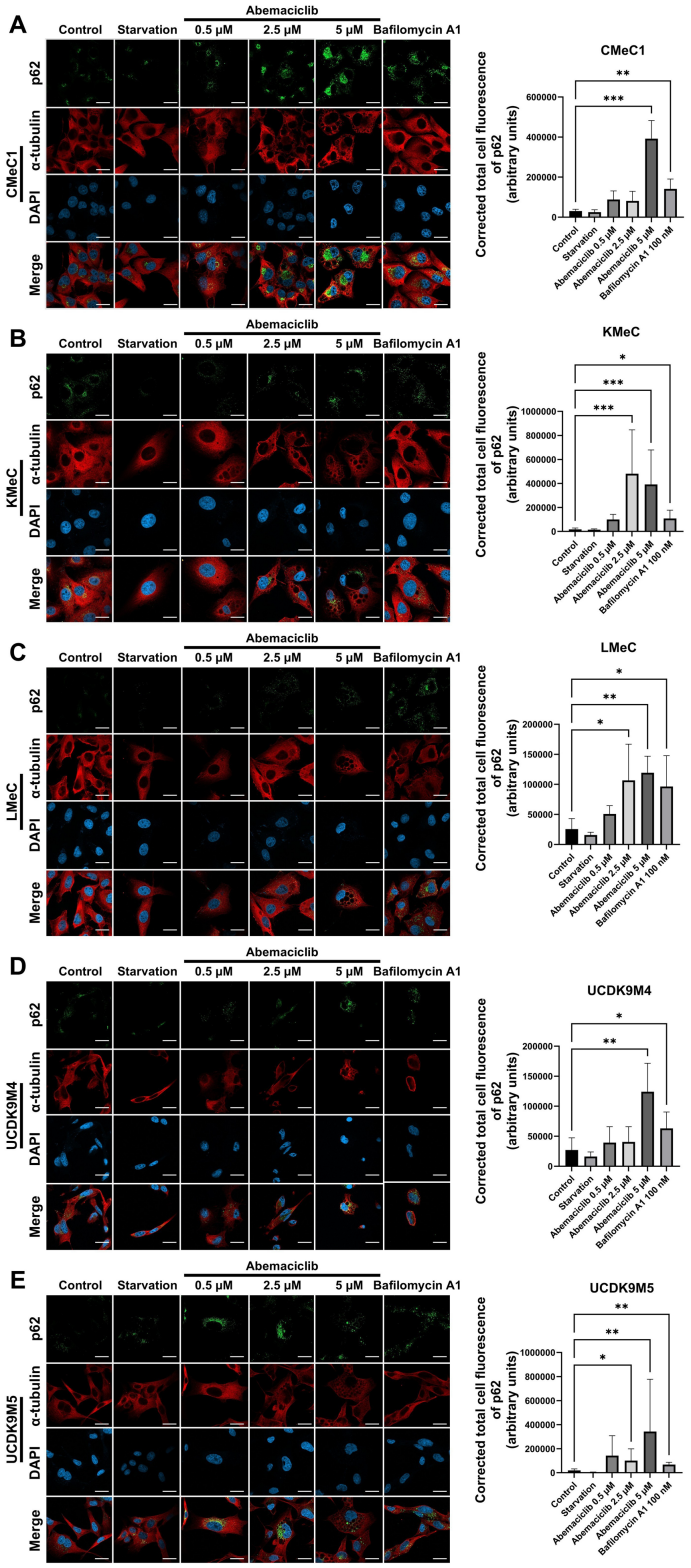
Immunofluorescence analysis of p62 accumulation in various canine melanoma cell lines after abemaciclib treatment. **(A–E)** Representative immunofluorescence images (left) and quantification of p62 intensity (right): CMeC1 **(A)**, KMeC **(B)**, LMeC **(C)**, UCDK9M4 **(D)**, and UCDK9M5 **(E)**. Cells were treated with abemaciclib (0.5, 2.5, or 5 μM) for 24 h, starved, or treated with bafilomycin A1 (100 nM) as a positive control for autophagy inhibition. p62 (green) was detected via immunofluorescence, α-tubulin (red) was used to visualize the cytoskeleton, and nuclei were counterstained with DAPI (blue). Merged images are shown in the bottom row of each set. Quantification of p62 levels was performed by measuring the corrected total cell fluorescence (CTCF) of p62 staining using ImageJ, expressed in arbitrary units (a.u.). Data represent mean ± SD from ≥3 independent fields. Statistical significance was determined using the Kruskal–Wallis test (^*^p < 0.05, ^**^p < 0.01, and ^***^p < 0.001). Scale bar = 20 μm.

To further evaluate autophagic completion, p62 levels were analyzed using immunofluorescence analysis (Figures 6A–E). p62, also known as sequestosome 1 (SQSTM1), is a selective substrate of autophagy that is degraded in autolysosomes (39). Therefore, its accumulation reflects impaired autophagic degradation. p62 levels increased significantly in all cell lines after 24 h treatment with 5 μM abemaciclib or 100 nM bafilomycin A1, indicating impaired autophagic degradation. Vacuolization was also noted under these conditions, further supporting the presence of lysosomal dysfunction. In KMeC, LMeC, and UCDK9M5 cells, 2.5 μM abemaciclib also significantly increased p62 levels (Figures 6B,C,E). At the lowest dose (0.5 μM), an increase in p62 intensity was observed, although the difference was not statistically significant.

### 3.7 Synergistic effect of abemaciclib and fenbendazole

**Figure 7:**
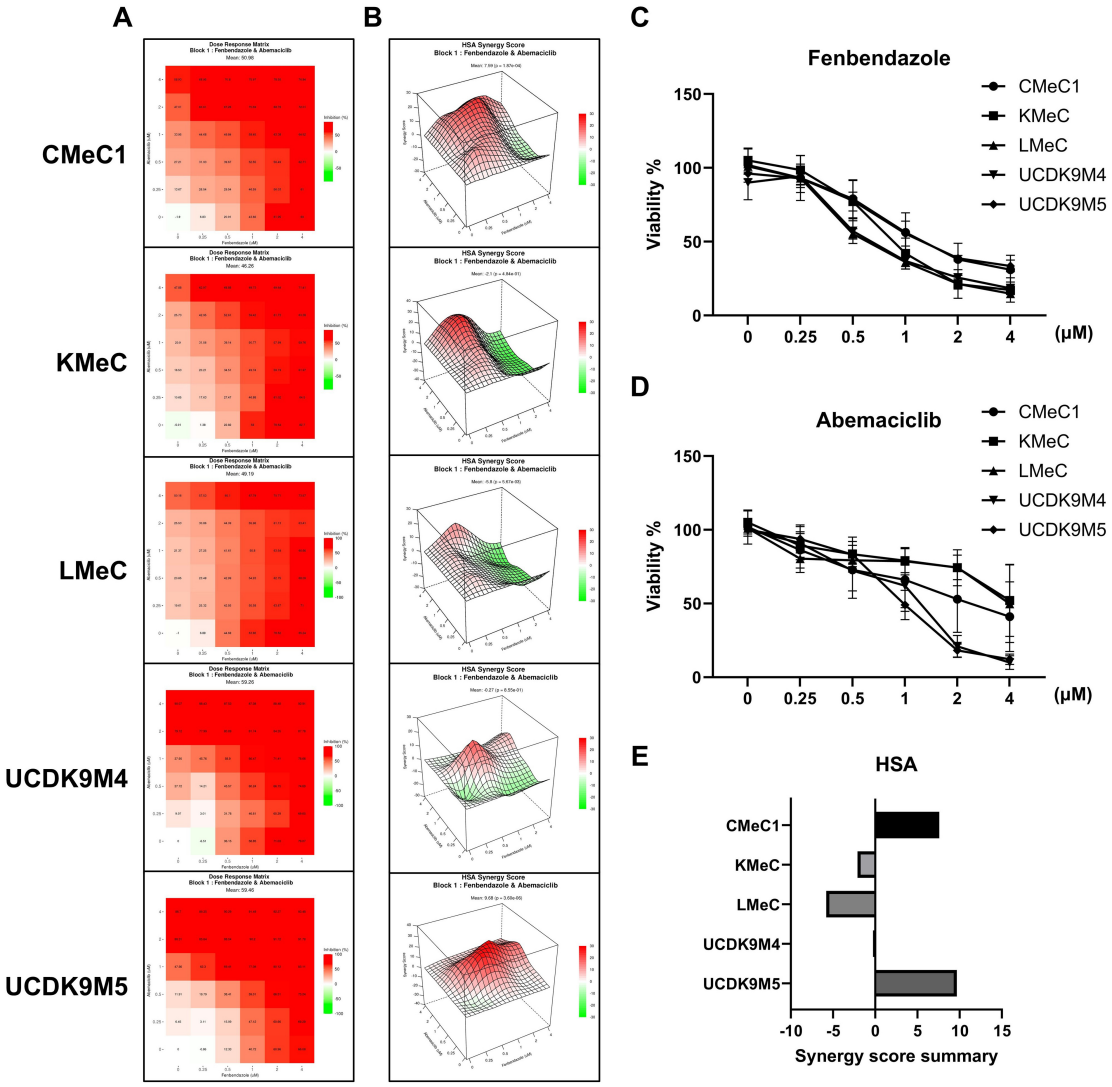
Synergistic effects of abemaciclib and fenbendazole on cell viability in canine melanoma cell lines. **(A)** Heatmaps showing combinational effects of abemaciclib and fenbendazole. Heatmaps illustrate the combinational effects of abemaciclib and fenbendazole on cell viability across various concentrations. Red indicates strong inhibition, whereas lighter shades represent reduced inhibition. **(B)** 3D surface plots illustrating the highest single agent (HSA) synergy scores. The HSA synergy score distribution for combination treatments of abemaciclib and fenbendazole is visualized in 3D plots for each cell line. Positive scores (red) indicate synergistic effects, whereas negative scores (green) indicate antagonistic effects. **(C,D)** The dose-dependent effects of abemaciclib and fenbendazole were measured in response to increasing concentrations of each drug as part of the combination treatment matrix. Data are presented as mean ± SD (*n* = 6), obtained from two replicates across three independent experiments. **(E)** Synergy score summary for each cell line. The bar graph summarizes the HSA synergy scores for the combination of abemaciclib and fenbendazole in each cell line. Positive scores indicate synergistic interactions, scores near zero suggest additive effects, and negative scores indicate antagonism. Among the cell lines, CMeC1 and UCDK9M5 demonstrated the highest synergy scores. HSA, highest single agent.

In a previous study, fenbendazole induced G2/M arrest and mitotic catastrophe in canine melanoma cells (40). To determine whether combining a cell cycle inhibitor targeting G1 arrest (abemaciclib) and G2/M arrest (fenbendazole) could produce a synergistic effect (41), the two drugs were tested in combination, and the HSA synergy score was calculated (Figures 7A,B). In single-dose treatments, fenbendazole showed a consistent dose-dependent viability decrease across all cell lines (Figure 7C), abemaciclib exhibited variable effects depending on the cell line: UCDK9M4 and UCDK9M5 showed low viability at concentrations higher than 1 μM compared to CMeC1, KMeC, and LMeC (Figure 7D). The HSA synergy analysis (Figure 7B) revealed dose-specific synergism between the two drugs. In CMeC1 and KMeC cells, a linear pattern of synergy scores was observed. Specifically, CMeC1 demonstrated synergism at fenbendazole doses below 2 μM, while KMeC showed synergism at doses below 1 μM. In contrast, UCDK9M4 and UCDK9M5 exhibited peak synergy scores at intermediate doses of both abemaciclib and fenbendazole, suggesting an optimal combination range for synergy. The Synergy Score Summary (Figure 7E) highlights cell line-specific responses. CMeC1 and UCDK9M5 showed high overall synergy scores, while KMeC and LMeC displayed negative synergy scores.

### 3.8 Combination of abemaciclib and fenbendazole suppresses canine melanoma growth in a mouse xenograft model

**Figure 8:**
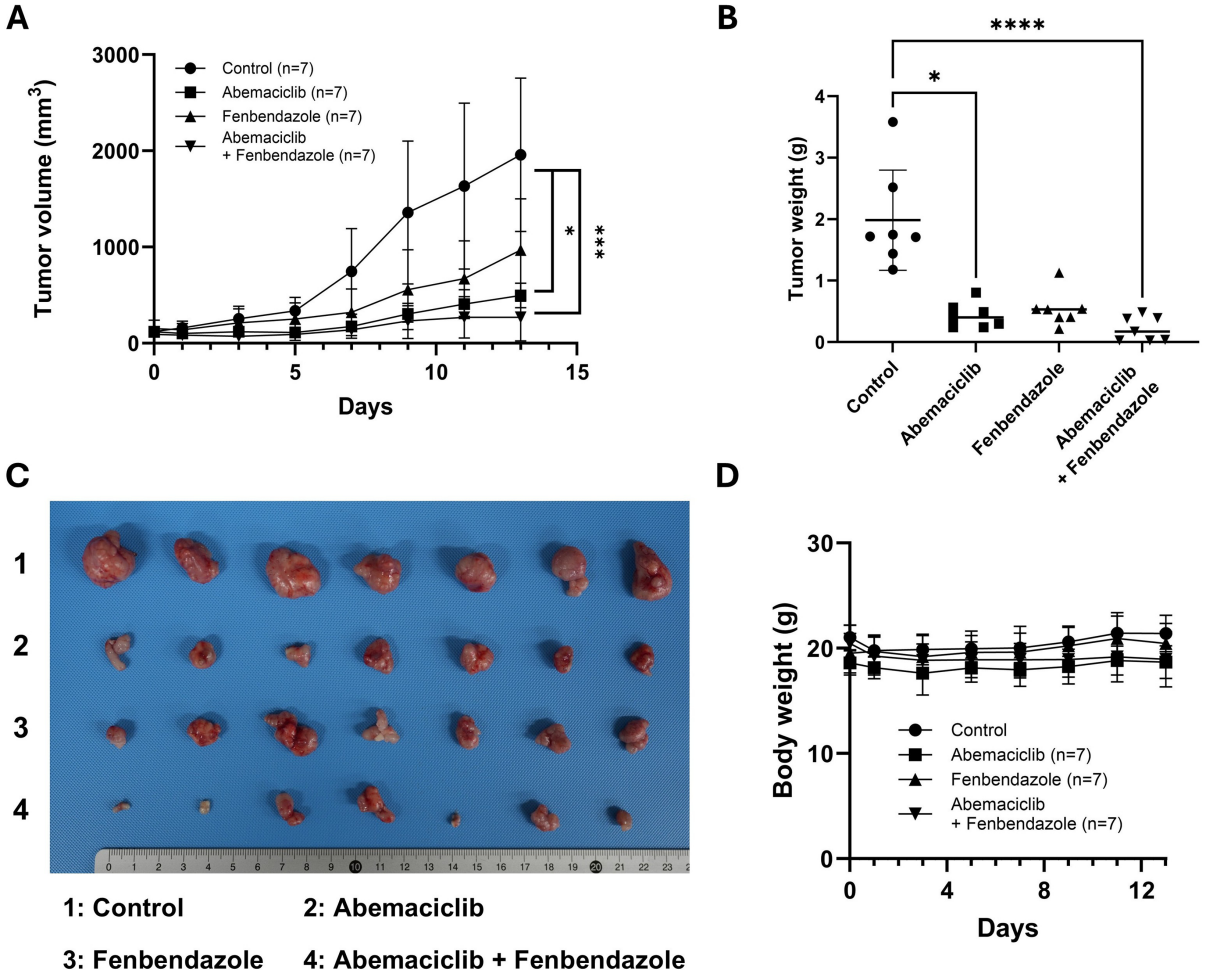
*In vivo* effects of abemaciclib and fenbendazole on tumor growth in a UCDK9M5 xenograft mouse model. **(A)** Tumor volume over time. UCDK9M5 cells were xenografted into BALB/c nude mice, and tumor volumes were measured over 13 days of treatment with control (vehicle), abemaciclib (50 mg/kg), fenbendazole (50 mg/kg), or a combination of abemaciclib and fenbendazole. The combination treatment significantly reduced tumor volume compared to the control and single treatments. Data are presented as mean ± SD (*n* = 7 per group). Statistical significance was determined using the Kruskal–Wallis test (^*^p < 0.05 and ^***^p < 0.001). **(B)** Tumor weight at the end of the experiment (day 13). The combination of abemaciclib and fenbendazole resulted in a significant reduction in tumor weight compared to the control and single treatments. Data are presented as mean ± SD (*n* = 7 per group). Statistical significance was determined using the Kruskal–Wallis test (^*^p < 0.05 and ^****^p < 0.0001). **(C)** Representative tumor images. Tumors excised at the endpoint are shown for each treatment group: 1: control, 2: abemaciclib, 3: fenbendazole, and 4: abemaciclib + fenbendazole. The combination treatment group shows visibly smaller tumors compared to the other groups. **(D)** Body weight changes during treatment. The body weights of mice were monitored throughout the experimental period to assess treatment-related toxicity. There were no significant differences in body weight among the groups, indicating that the treatments were well tolerated (*n* = 7 per group).

In our previous study, the anti-tumor effects of fenbendazole were demonstrated in canine melanoma cells (40), whereas we investigated the effects of abemaciclib in this study. To validate the therapeutic potential of these drugs, either alone or in combination, a mouse xenograft model was established. UCDK9M5 cells were injected into BALB/c nude mice. Mice were treated with vehicle (control), abemaciclib, fenbendazole, or their combination for 13 days (Figure 8A). Treatment with abemaciclib alone or combination with fenbendazole significantly reduced tumor volume compared to the control group (Figure 8A). Although fenbendazole treatment also led to a reduction in tumor volume, the difference did not reach statistical significance. After euthanasia, the tumor weight was measured, which showed a significant reduction in the abemaciclib and combination treatment groups compared to that of the control (Figures 8B,C). Notably, tumors from the combination group appeared visibly smaller than in the control, suggesting a potential additive or synergistic effect (Figure 8C). Notably, no significant body weight loss or adverse effects were observed in any treatment group, indicating that the treatments were well-tolerated (Figure 8D).

## 4 Discussion

In the previous study, long-term exposure to the CDK4/6 inhibitor palbociclib also demonstrated enhanced antiproliferative effects at lower concentrations, primarily through cytostatic growth inhibition rather than acute cytotoxicity, particularly in estrogen receptor-positive breast cancer cell lines (42). This supports the notion that prolonged treatment with CDK4/6 inhibitors may exert greater tumor-suppressive effects compared to short-term assays. In this study, short-term viability assays (CCK-8, 3 days, Figure 1B) and long-term proliferation assays (colony forming assay, 8 days, Figure 1D) demonstrated distinct IC_50_ values, suggesting that long-term treatment with abemaciclib at lower concentrations effectively suppresses the proliferative capacity and survival of melanoma cell lines compared to acute cytotoxicity assessments. A previous study reported that trough plasma concentration of approximately 200 ng/mL of abemaciclib is required to maintain cell cycle arrest (43). Although pharmacokinetic data of abemaciclib in dogs are currently unavailable, findings from the colony formation assay suggest that a chronic dosing may be sufficient to achieve comparable therapeutic exposure. During the cell viability assay, treatment with abemaciclib at concentrations above 2.5 μM resulted in distinct morphological changes.

In anti-cancer treatments, senescence is treatment with abemaciclib above 2.5 μM induced morphological changes. These morphologic changes are hallmark characteristics of cellular senescence, which are often associated with a flattened and enlarged morphology, cytoplasmic vacuolization, and the occasional presence of multiple or enlarged nuclei (44). Senescence in the context of anti-cancer therapy has dual characteristics. It is typically defined as a stable and long-lasting arrest in the cell cycle of surviving tumor cells, and it can occur in non-malignant cells (45). However, therapy induced or oncogene-derived senescence activates senescence-associated secretory phenotype (SASP), secretion of the inflammatory cytokine. This secretory phenotype can reinforce of the senescent state and also promote the recruitment of M1-like macrophages (46).

Notably, in UCDK9M5 cells, a dose-dependent effect on cell cycle distribution was observed. Treatment with 0.5 and 1 μM abemaciclib resulted in an increased proportion of cells in the G1 phase. However, at higher concentrations (2 and 4 μM), the G2/M phase population was restored (Figure 3B), accompanied by notable changes in histogram morphology compared to that of the control (Figure 3A). To investigate this phenomenon, morphologic staining was performed (Supplementary Figure 1), and the number of multinucleated or macronucleated cells was counted. However, no significant differences were observed when compared to other cell lines.

Regarding this phenomenon, a previous study has demonstrated that the loss of CDK4/6 activity during the S/G2 phase can disrupt normal mitosis (47). This occurred because G2-phase cells were unable to enter mitosis and became arrested. As a result, the cells showed accumulation of DNA content and entered to a G0-like state. Furthermore, other reports have pointed out a non-traditional role of CDK4 in contributing to G2/M arrest under specific stress conditions (48). These mechanisms may help explain the accumulation of cells in G2/M phase at higher concentrations of abemaciclib in UCDK9M5 cells. However, further studies are required to clarify this hypothesis.

All cells treated with concentrations exceeding 2.5 μM exhibited prominent vacuolization. Vacuolization is associated with various cellular processes, including endocytosis, methuosis (arising from macropinocytosis) (49), paraptosis (triggered by ER stress) (50), autophagy (51), senescence (44), lysosomal dysfunction (31), and osmotic stress-induced cell swelling (52). Lysosomal dysfunction, characterized by swollen and dysfunctional lysosomes, have been reported (31, 53). Furthermore, lysosome-derived vacuoles contained undigested debris and dysfunctional lysosomes (31). In this study, we demonstrated that the vacuolization originated from lysosomes. This conclusion was supported by FITC-dextran study, which ruled out extracellular components (Figures 5A, 6A), and the effective suppression of vacuolization by V-ATPase inhibitors. Additionally, organelle remnants were observed within vacuoles (Supplementary Video 1).

In western blot analysis, abemaciclib treatment effectively inhibited RB phosphorylation in all canine melanoma cell lines. In its hypophosphorylated state, RB suppresses the transcription of genes essential for cell cycle progression. This suppression occurs through interaction with the transactivation domain of the E2F transcription factor family (15). Additionally, Cyclin A2 expression decreased in a dose-dependent manner across all cell lines. Given that Cyclin A2 transcription is predominantly regulated by E2F activity (54), this reduction is likely attributable to RB hypophosphorylation. In contrast, cleaved PARP, a marker of apoptosis, exhibited only weak signal intensity following abemaciclib treatment, suggesting that apoptosis may not be a major mechanism of cell death under the tested conditions. Senescent cells are known to be resistant to both extrinsic and intrinsic apoptosis (55). Therefore, these findings suggest that the cell death mechanisms in these cells involve senescence and lysosomal dysfunction, rather than apoptosis.

Furthermore, Cyclin D1 expression was found to be increased in all cell lines. Cyclin D1 is regulated by the AP-1 (activator protein-1) transcription factor family and its activation involves multiple signaling pathways, including PI3K/AKT/mTOR, MAPK, STAT, NF-κB, Wnt/β-catenin, estrogen receptors, progesterone receptors, and androgen receptors (56, 57). Cyclin D1 upregulation may represent a compensatory response to RB pathway inhibition.

Non-steroidal aromatase inhibitors, either alone or in combination with CDK4/6 inhibitors, are the first-line treatment options for human breast cancer (HR^+^/HER2^−^) (58). Cyclin D1 is a major transcriptional target of the estrogen receptor, and its expression is inhibited by non-steroidal aromatase inhibitors (58). Therefore, targeting Cyclin D1 may offer an additional therapeutic strategy to suppress cell cycle progression in canine melanoma cells.

Differential expression patterns of Cyclin E1 were observed between abemaciclib-resistant (KMeC and LMeC) and -sensitive cell lines (CMeC1, UCDK9M4, and UCDK9M5), suggesting a potential link to resistance mechanisms. Cyclin E1 synthesis is regulated by the E2F-DP complex, which is activated following RB phosphorylation by CDK4/6-Cyclin D complex (59). Although all cell lines efficiently suppressed p-RB upon abemaciclib treatment, resistant cell lines (KMeC and LMeC) exhibited increased Cyclin E1 expression. Furthermore, Cyclin E overexpression can facilitate resistance to CDK4/6 inhibitors (60, 61). Accordingly, Cyclin E1 overexpression in canine melanoma cells may similarly contribute to abemaciclib resistance.

CDK4/6 inhibitors induced autophagy in multiple myeloma cells (abemaciclib) (62), fibroblasts, and leukemia cells (palbociclib) (63). Measuring changes in LC3 is the most common method to quantify autophagy, as it reflects the dynamic conversion of LC3-I (cytosolic form) to LC3-II (membrane bound form) during the autophagic process (64). Autophagy progresses through distinct steps, including initiation, elongation, autophagosome formation, and autolysosomes generation (via fusion with lysosomes) (65). During autophagosome formation, LC3-II level increases as LC3-I is conjugated to phosphatidylethanolamine and incorporated into the autophagosomal membrane. Conversely, LC3-I levels decrease as this conversion occurs. However, LC3-II is subsequently degraded during autolysosome formation and the completion of autophagic degradation (65).

A previous study monitoring autophagic flux using a fluorescence probe demonstrated that abemaciclib treatment results in LC3 accumulation, indicating autophagic flux inhibition (31). In our study, autophagy completion was assessed using immunofluorescence analysis of p62. At high concentrations, abemaciclib caused significant p62 accumulation as in the case of bafilomycin A1 treatment. These observations, including p62 accumulation and vacuolization, collectively suggest that abemaciclib induces lysosomal dysfunction and impairs autophagy completion in a dose-dependent manner.

To summarize the cellular outcomes identified in this study, cancer cell proliferation was inhibited primarily through G1 phase arrest and senescence. Moreover, although autophagy was initiated, its completion was impaired due to lysosomal dysfunction, which suggests a blockage in autophagic flux. Therefore, abemaciclib causes a distinct cellular phenotype characterized by senescence and lysosomal dysfunction. These findings provide mechanistic insight into the cytostatic effects of abemaciclib and highlight the therapeutic relevance of targeting the autophagy-lysosome axis in combination strategies. Previous studies have demonstrated that targeting the autophagy-lysosome axis can induce cytotoxic effects and non-canonical cell death (31, 66). Moreover, combination with lysosomotropic agents has been proposed as a novel treatment strategy (67). Therefore, the use of abemaciclib may offer a new treatment option because it induces G1 cell cycle arrest and simultaneously targets lysosomal dysfunction.

Co-treatments using G1 and G2/M phase arrest agents induce antagonistic effects, possibly due to conflicting mechanisms of cell cycle regulation (68). However, sequential treatment strategies have synergistic effects (69–71), and some studies have even demonstrated enhanced cytotoxic effects with co-treatment (72). In our previous study (40), we observed that fenbendazole treatment in canine melanoma cells induced G2/M arrest and subsequent mitotic slippage, which was characterized by multinucleated and macronucleated cells. Based on these findings, we hypothesized that combining cell cycle inhibitors targeting the G1 phase could effectively target mitotic slippage cells and enhance senescence effects. Considering the practical challenges of drug administration, such as optimizing medication scheduling and achieving effective pharmacokinetic plasma concentrations, we selected a co-treatment approach rather than sequential treatment strategy. This decision was based on the oral administration characteristics of both fenbendazole and abemaciclib, which differ from those of the intravenous drugs used in previous studies (69, 70). The synergy scoring test revealed that co-treatment exhibited synergistic effects within specific concentration ranges. Notably, cells with high sensitivity to abemaciclib demonstrated high synergistic scores.

In the *in vivo* study, fenbendazole treatment alone reduced tumor volume and weight; however, this reduction was not statistically significant. This outcome may be attributed to the high variability in tumor size within the fenbendazole-treated group, as well as its limited efficacy as a monotherapy. In contrast, the combination therapy produced a substantial and consistent reduction in tumor burden. These findings highlight the therapeutic potential of combining cell cycle inhibitors targeting different phases.

In summary, our findings indicate that combining cell cycle inhibitors that target different phases can produce synergistic effects. In this study, we evaluated the use of abemaciclib for the first time in canine melanoma cells, and demonstrated its ability to induce autophagy, senescence, and distinct cytoplasmic vacuolization features. Furthermore, combination therapy offers a novel therapeutic strategy to enhance senescence induction using complementary cell cycle inhibitors.

## Data Availability

The original contributions presented in the study are included in the article/Supplementary material, further inquiries can be directed to the corresponding author.
