# Supplementary material for: Abemaciclib induces G1 arrest and lysosomal dysfunction in canine melanoma cells: synergistic effects with fenbendazole
Source: Front Vet Sci. 2025 Jun 26;12:1603686. doi: 10.3389/fvets.2025.1603686 (PMC12240792; doi:10.3389/fvets.2025.1603686)
Supplement: Supplementary file 1 [file Table_1.docx]

| Antibody | Species | Manufacturer (Catalog#) | Dilution |
| --- | --- | --- | --- |
| RB | Rabbit | Abclional (A16966) | 1:1000 |
| p-RB (S807/811) | Rabbit | Abclonal (AP0484) | 1:1000 |
| CCNA2 | Rabbit | Abclonal (A7632) | 1:1000 |
| CCNE1 | Rabbit | Abclonal (A14225) | 1:1000 |
| CCND1 | Rabbit | Abclonal (A19038) | 1:1000 |
| Cleaved PARP | Rabbit | Abclonal (A19612) | 1:2000 |
| LC3 B | Rabbit | Abclonal (A19665) | 1:1000 |
| GAPDH | Mouse | Abclonal (AC002) | 1:15,000 |

**Supplementary Table 1. List of primary antibodies used for western blot analysis**
