# Supplementary material for: Abemaciclib induces G1 arrest and lysosomal dysfunction in canine melanoma cells: synergistic effects with fenbendazole
Source: Front Vet Sci. 2025 Jun 26;12:1603686. doi: 10.3389/fvets.2025.1603686 (PMC12240792; doi:10.3389/fvets.2025.1603686)
Supplement: Supplementary file 2 [file Table_2.docx]

| Gene name | Gene symbol | Primer direction | Primer (5’-3’) | GenBank accession no. | Amplicon size |
| --- | --- | --- | --- | --- | --- |
| *Cyclin A2* | CCNA2 | *Sense* | TGACCTAGCTGCACCAACAG | XM_038425855.1 | 205 |
| *Cyclin A2* | CCNA2 | *Anti-sense* | CCAGCTCTGTCCTGTGACTG |  |  |
| *Cyclin B1* | CCNB1 | *Sense* | GAAGATCGGTATGGCAGGGG | XM_038441478.1 | 126 |
| *Cyclin B1* | CCNB1 | *Anti-sense* | TTGGCCTGTGGTTGTTCACT |  |  |
| *Cyclin E1* | CCNE1 | *Sense* | TCGCAGAGCTTTTGGATCTT | XM_038529535.1 | 165 |
| *Cyclin E1* | CCNE1 | *Anti-sense* | GCACCATCCACTTGACACAC |  |  |
| *Cyclin E2* | CCNE2 | *Sense* | CGCAGTAGCCGTTTACAAGC | XM_072803861.1 | 102 |
| *Cyclin E2* | CCNE2 | *Anti-sense* | CTGGGCGGTTTTCCTCTTCT |  |  |
| *Cyclin D1* | CCND1 | *Sense* | CCAGTGGCAGAGGAGAACAA | XM_025451759.3 | 149 |
| *Cyclin D1* | CCND1 | *Anti-sense* | CCCAGGTGTAGACCTTGCAC |  |  |
| *Cyclin-dependent-kinase 4* | CDK4 | *Sense* | CCCCGTCCAGTACAGACAGT | XM_038679138.1 | 100 |
| *Cyclin-dependent-kinase 4* | CDK4 | *Anti-sense* | AGGCAGAGATTCGCTTGTGT |  |  |
| *Cyclin-dependent-kinase 6* | CDK6 | *Sense* | AGGGCATGCCGCTCTCCACCATCC | XM_038556826.1 | 353 |
| *Cyclin-dependent-kinase 6* | CDK6 | *Anti-sense* | GATGCGGGCAAGGCCGAAGTCAGC |  |  |
| *Glyceraldehyde-3-phosphate dehydrogenase* | GAPDH | *Sense* | AACATCATCCCTGCTTCCAC | XM_072819089.1 | 234 |
| *Glyceraldehyde-3-phosphate dehydrogenase* | GAPDH | *Anti-sense* | GACCACCTGGTCCTCAGTGT |  |  |

**Supplementary Table 2. Primer sequence used for gene amplification in canine cel lines**
