# Supplementary figures and images for: Abemaciclib induces G1 arrest and lysosomal dysfunction in canine melanoma cells: synergistic effects with fenbendazole
Source: Front Vet Sci. 2025 Jun 26;12:1603686. doi: 10.3389/fvets.2025.1603686 (PMC12240792; doi:10.3389/fvets.2025.1603686)

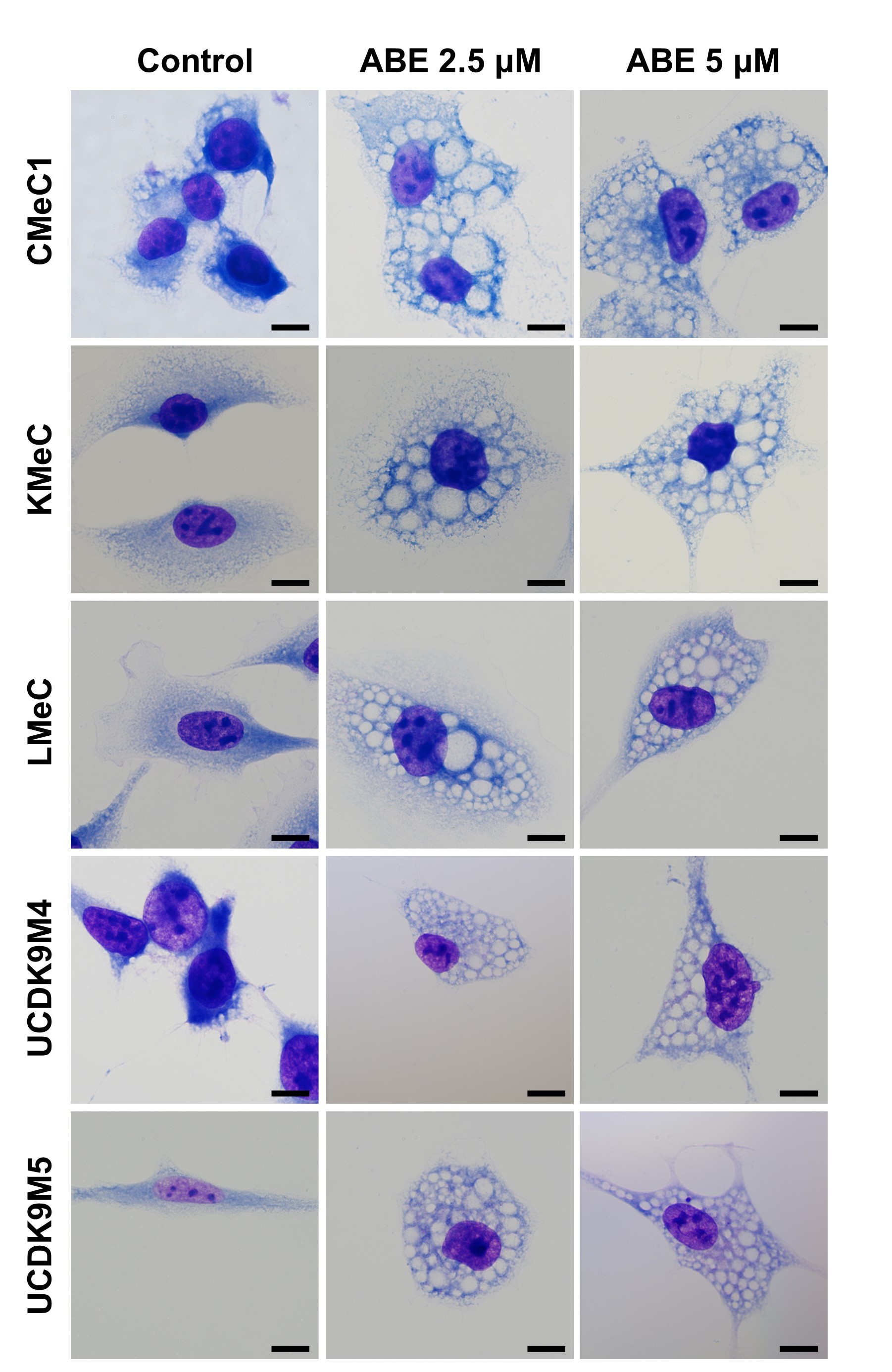

Supplement: Supplementary file 4 [file Image_1.jpeg]
